# Myelin and Modeling: Bootstrapping Cortical Microcircuits

**DOI:** 10.3389/fncir.2019.00034

**Published:** 2019-05-08

**Authors:** Robert Turner

**Affiliations:** ^1^Department of Neurophysics, Max Planck Institute for Human Cognitive and Brain Sciences, Leipzig, Germany; ^2^Sir Peter Mansfield Imaging Centre, University of Nottingham, Nottingham, United Kingdom; ^3^Spinoza Centre for Neuroimaging, University of Amsterdam, Amsterdam, Netherlands

**Keywords:** brain, myelin, MRI, cortical microcircuitry, develoment, modeling

## Abstract

Histological studies of myelin-stained sectioned cadaver brain and *in vivo* myelin-weighted magnetic resonance imaging (MRI) show that the cerebral cortex is organized into cortical areas with generally well-defined boundaries, which have consistent internal patterns of myelination. The process of myelination is largely driven by neural experience, in which the axonal passage of action potentials stimulates neighboring oligodendrocytes to perform their task. This bootstrapping process, such that the traffic of action potentials facilitates increased traffic, suggests the hypothesis that the specific pattern of myelination (myeloarchitecture) in each cortical area reveals the principal cortical microcircuits required for the function of that area. If this idea is correct, the observable sequential maturation of specific brain areas can provide evidence for models of the stages of cognitive development.

## Introduction

Mammalian cerebral cortex invariably shows localized functional specialization, such that the performance of specific actions is associated with specific and reproducible patterns of increased and decreased neural activity on a spatial scale of a few millimetres, often organized in larger scale networks across the brain. Anatomically, the cortex comprises relatively distinct areas, on a similar same spatial scale, each with quite uniform cytoarchitecture and myeloarchitecture. These areas form the basis of parcellations such as those of Brodmann (1909), Vogt and Vogt (1919) and many more recent studies (reviewed in Caspers et al., 2013), amounting in human brain to an inventory of perhaps 200 cortical areas which are distinguishable in every normal brain. Studies of chemoarchitecture (reviewed in Palomero-Gallagher and Zilles, 2018), using staining and autoradiographic techniques specific to particular neuroreceptors, also reveal sharply bounded cortical areas. For some simple tasks, such as visual (Bridge et al., 2005) and auditory (Dick et al., 2012; De Martino et al., 2015) perception, good congruence has been observed between the anatomically and functionally defined boundaries of the relevant cortical areas, and these boundaries often coincide with chemoarchitectonic boundaries. In describing their hybrid cortical parcellation scheme, Glasser and Van Essen (2011) furthermore assert that the boundaries they observe, using a crude magnetic resonance imaging (MRI) measure of cortical myelination, coincide with those they derive from maps of functional connectivity in the so-called “resting state,” in those cortical regions where both types of boundary are clearly definable. For a more quantitative MRI measure of myelin, see Geyer et al. (2011), Stüber et al. (2014) and Callaghan et al. (2015).

The simple question naturally arises: what is special about each cortical area? Putting it another way, does each cortical area have an identifiable particular processing competence? The idea is by no means new that brain function can be explained mechanistically at the systems level (for example, Logothetis et al., 2010; Turner, 2016) and considerable progress has recently been made, with such concepts as voxel encoding (Naselaris et al., 2011) and representational similarity (Diedrichsen and Kriegeskorte, 2017) in using functional MRI (fMRI) experiments to identify feature spaces that specific neural architecture may be responsible for parsing.

When axonal fibers have been tracked using chemical tracing techniques, or diffusion-weighted MRI, it has been established that the connectivity of some areas is dominated by a particular set of input or output pathways (Felleman and Van Essen, 1991). However, recent work by Han et al. (2018) has demonstrated that even in primary visual cortex, the most strikingly specialized cortical area, neurons make a wide range of connections to other cortical and subcortical regions. It would be surprising if other cortical areas had simpler connectivity. Such repertoires of long-range connections shared as a connectional fingerprint (Gorbach et al., 2011) within any particular area and established during the later stages of gestation, may explain the relative uniformity of cellular architecture within each cortical area. The cytoarchitecture characteristic of a particular cortical area is also genetically prescribed during early development (Burt et al., 2018; Gomez et al., 2018; Wen et al., 2018), as neuronal cell bodies of various types find their layer-dependent destinations at the end of their long traverse from their birthplaces. Recent work by Burt et al. (2018) found a close resemblance between MRI-based myelin maps of the cortex (which reliably index the cortical functional hierarchy) and the dominant pattern of transcriptomic variation across the cortex. Gradients in the profiles of gene expression could be related to microcircuit function and neuropsychiatric disorders.

But the functionality of neurons within a cortical area must critically depend on the pattern of connections local to that area—connections which are formed, re-formed and consolidated throughout an organism’s life—in other words, its microcircuitry. While for some purposes a “canonical microcircuit” (Shipp, 2007) can crudely represent the intracortical wiring diagram, there is considerable diversity of cyto- and myeloarchitecture across the cortex, which may well provide a key to much improved modeling of brain function.

At stake is a continuing controversy in systems neuroscience, with qualitatively different approaches to cognitive modeling and its plausibility in regard to empirical evidence. In a recent article, Turkheimer et al. (2019) argue as follows. “Drawing on the epistemological literature, we observe that due to their loose mechanistic links with the underlying biology, models based on strong forms of emergence (such as those of Friston, using the idea of free energy) are at risk of metaphysical implausibility. This, in practical terms, translates into the over-determination that occurs when the proposed model becomes only one of a large set of possible explanations for the observable phenomena. On the other hand, computational models that start from biologically plausible elementary units, hence are weakly emergent, are not limited by ontological faults and, if scalable and able to realistically simulate the hierarchies of brain output, represent a powerful vehicle for future neuroscientific research programmes.” Research springing from the proposals in the present article may provide a firmly grounded ontology for realistic modeling of brain function.

I address the question whether cortical microcircuits can be defined and differentiated using the mesoscopic tools of MRI, and thus investigated as they develop *in vivo*. The argument rests on the key histological observations of Smith (1907), Vogt and Vogt (1919), Flechsig (1920), and those of more recent researchers using MRI to quantitatively assess the distribution of myelin in the cerebral cortex (Geyer et al., 2011; Glasser and Van Essen, 2011; Lutti et al., 2014). The fundamental points are: (a) myelination is generally driven by a neuron’s experience; (b) it is only once the relevant axons have reached an appropriate level of myelination that cortical microcircuits can be considered to be consolidated, and thus available for routine later use; and (c) the distribution of myelin in the white matter and cerebral cortex is observable *in vivo* with a spatial resolution of about 300 microns using MRI techniques at high magnetic field.

## Benefits of Myelination

It is worth listing the advantages conferred by the myelination of axons, an expedient evolved hundreds of millions of years ago and first noted in cartilaginous fish.

Perhaps most importantly, myelination speeds up the conduction velocity of action potentials by 10–50-fold for an axon of a given diameter, by enabling salutatory conduction (reviewed in Nave and Werner, 2014). It also makes transmission of action potentials more efficient, increases robustness of axons (Staal and Vickers, 2011), enhances the arrival synchrony of action potentials, reduces electrical crosstalk between axons and other nearby neurites (Hull et al., 2015), enables neural regeneration after damage (Jessen and Mirsky, 2016), and, last but not least, myelination suppresses synaptic plasticity in gray matter by inhibiting neurite growth (Lozano et al., 1995; Thallmair et al., 1998) and preventing the formation of synaptic contacts on already-myelinated axons (Braitenberg, 1962).

It is this final property of myelination, well discussed by Glasser et al. (2014), that is of greatest relevance in the present work. It is reasonable to suppose that in gray matter the axons that become myelinated are those which are most important in providing dedicated cortical microcircuits. Myelination confers durability and reliability, the basic requirements for the observed specialization of function. Study of the variations in myeloarchitecture across the cortex may thus provide unique insights into the specific competencies of different cortical areas. Before such variations are discussed, some points need to be made regarding how myelination comes about.

## The Process of Myelination

The main networks of peripheral nerves and neurons are established during gestation, but in humans at birth very few of the axons in the brain are already myelinated (Flechsig, 1920), mostly in the optic radiation and the cerebrospinal tract. Brain tissue is rich in oligodendrocyte precursor cells, which transform into mature oligodendrocytes when appropriately stimulated, and proceed to wrap adjacent axons with a sheet of myelin (Snaidero et al., 2014), which may be up to about 16 layers thick. For a given oligodendrocyte and local stretch of axon, this process takes about 5 h (Czopka et al., 2013) and the oligodendrocyte involved generally continues to support the myelin sheath that it has generated until the neuron concerned dies from damage or old age (Yeung et al., 2014).

By adulthood, most white matter axons are myelinated, although a few unmyelinated axons can still be found even in early-myelinated axonal bundles such as the optic radiation, and some WM regions, such as the prefrontal lobes, are slow to mature in human brains (Giedd et al., 2015) reaching their plateau myelination level in people more than 30 years old (Yeatman et al., 2014). Short association fiber bundles, including U-fibers, are generally unmyelinated at birth and become myelinated in a well-defined order (Brody et al., 1987; Kinney et al., 1988). The topic of white matter maturation is well reviewed by Dubois et al. (2014). In a similar way, gray matter axons are progressively myelinated (Guillery, 2005; Lebenberg et al., 2019) cortical area by area.

Extensive recent research has focused on the mechanism of myelination, especially on its relationship to neural activity (well reviewed in Chorghay et al., 2018; Almeida and Lyons, 2017). Oligodendrocytes commence the myelination of a nearby axon when: (a) the axon has a larger diameter than roughly 0.5 micrometres (Nave and Werner, 2014); and/or (b) a sufficient number of action potentials have passed along the unmyelinated axon. The passage of action potentials itself can promote an increase of axon diameter (Costa et al., 2018) so that activity might be ultimately considered the primary driver of myelination. Once an axon is myelinated, several mechanisms are available to fine-tune the conduction velocity, a control needed to ensure the arrival-time synchrony of action potentials at synaptic destinations. These mechanisms include the axon diameter (Chéreau et al., 2017), myelin thickness (Rushton, 1951; Waxman, 1975; Campbell et al., 2018), length of continuous axonal segment that is wrapped (Rushton, 1951; Brill et al., 1977), as well as the nodal geometry itself (Arancibia-Cárcamo et al., 2017).

In the light of these findings, it can be fairly claimed that neural experience shapes the patterns of myelination that develop as a brain matures (Hill et al., 2018; Hughes et al., 2018; Stedehouder et al., 2018) and learns new skills (McKenzie et al., 2014; Xiao et al., 2016). It follows that the neural circuits which are thus preferentially constructed, as self-organizing networks, are requisite for the appropriate performance of the specific brain area where they are located. Because myelination protects the axons included in these circuits from additional synaptic connections and renders them more durable, their configuration may provide clues regarding the transformational operations that each brain area is equipped to perform.

## Myeloarchitecture

The surprisingly sparse histological literature on myeloarchitecture has been brilliantly reviewed by Nieuwenhuys (2013) and Nieuwenhuys et al. (2015). Much of the most careful and detailed histological work was performed by Oscar and Cecile Vogt and their team between 1905 and 1955, although a significant number of their publications have not yet been translated from their original German. By creating a feature-based terminology, the Vogts were able unambiguously to discriminate cortical areas from each other and claimed that the boundaries they discovered were sharp. This confirmed Elliot Smith’s observation (1907) that “there is a very widespread belief that the characters of one area merge gradually and imperceptibly into those of the neighboring areas, but this is entirely mistaken. The changes in structure occur with the utmost abruptness so that it is possible to determine with absolute precision the exact boundaries of each area. If the reader needs convincing of the accuracy of this statement let him cut a fresh brain at right angles to the calcarine, intraparietal (any part), central, inferior frontal, or parallel sulci, and he will find all the confirmation that is necessary.” Recent work by Nieuwenhuys et al. (2015) and Nieuwenhuys and Broere (2017) has transferred the drawings of myeloarchitectonic areas made by the Vogts and their team to the modern Montreal Neurological Institute template brain (Colin27).

**Figure 1:**
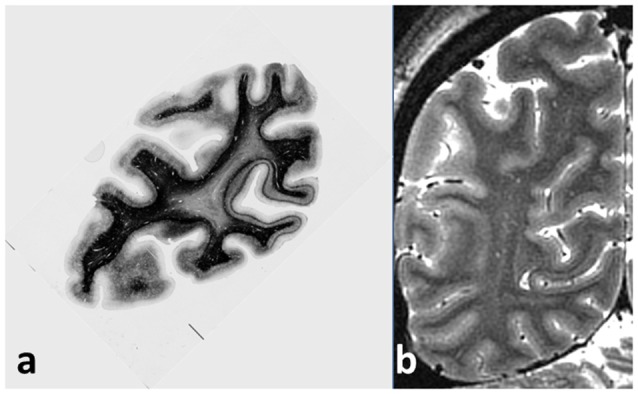
**(A)** Coronal section of cadaver human brain, stained for myelin using the Gallyas stain. The stria of Gennari, located in the calcarine sulcus, is easily visible on the right, and a cortical area with a pronounced band of Baillarger (probably MT/V5) appears at the left. Note the sharpness of the boundaries between areas with different myeloarchitecture, and the uniformity of the architecture within each area. **(B)** Coronal magnetic resonance imaging (MRI) section of live occipital human brain at 7T, GRASE imaging sequence, 0.5 mm isotropic voxels, obtained at the Leipzig Max Planck Institute for Human Cognitive and Brain Sciences. The same two cortical areas with a visible myelinated layer can easily be picked out, together with other areas also showing a band of Baillarger.

**Figure 2:**
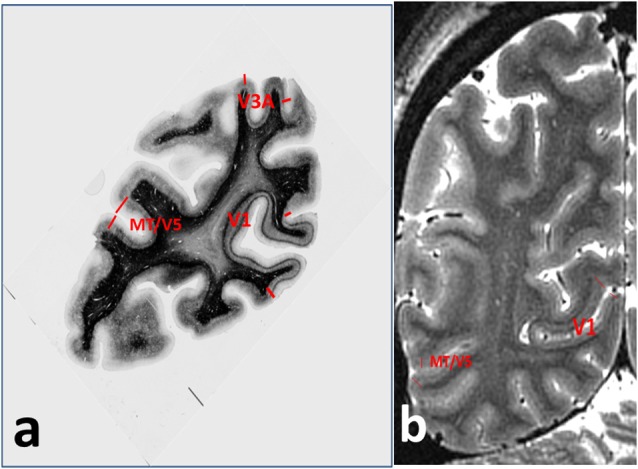
Annotations for Figure 1, showing **(A)** cortical areas V1, V3A and MT/V5 on the myelin-stained histological section, and **(B)** V1 and MT/V5 on the MRI section.

Even a cursory examination of a cadaver human brain section (Figure 1A) stained using the Gallyas technique (Gallyas, 1979) for myelin demonstrates the strength of the Vogts’ claim. Here, several distinct gray matter areas are easily distinguishable by eye, as described by Clarke and Miklossy (1990), with boundaries which are sharp compared with the cortical thickness, by the density and distribution of the cortical myelin—even allowing for the variations of the angle between the cutting plane and the cortical sheet. Figure 2A shows two cortical areas that are easily identifiable in this section. Lacking complete deformable 3D atlases of human brain myeloarchitecture, it is not yet possible to match these sections precisely.

**Figure 3:**
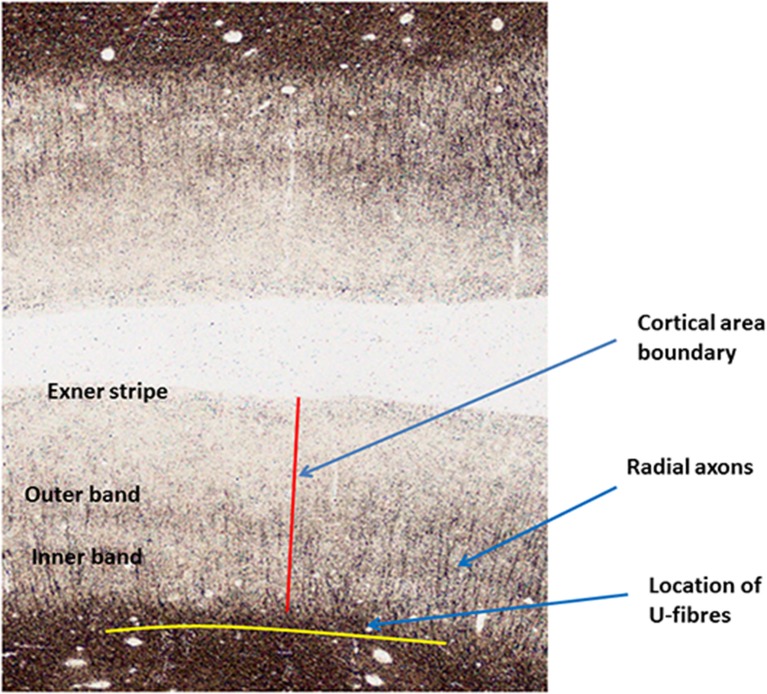
Annotated Gallyas-stained histological section of human parietal cortex. Positions are indicated for the Exner stripe, the bands of Baillarger, radial myelinated axons and U-fibers.

Most of the myelinated axonal fibers in gray matter are radial or horizontal (see Figure 3). Radial fibers emerge from the white matter with a myelin sheath that can extend as far as cytoarchitectonic Layer II (depending on cortical area). In some areas, such as motor cortex, these fibers are strikingly bunched into groups of 12–16 axons. Horizontal fibers are mostly clustered into the two bands of Baillarger (1840) lying in cortical Layers IV and V, and in the Exner stripe, in cortical Layer I (Lam and Sherman, 2018; Schuman et al., 2019). The bands of Baillarger, between 100 and 300 micrometres thick and often visible by naked eye in fresh cadaver brain without tissue staining (Smith, 1907), vary in density, thickness and depth, again depending on cortical area, while the thinner Exner stripe is remarkably uniform across the entire cortical surface.

It is a reasonable assumption that myelinated axons play the most important roles in local neural activity, due to their three advantageous properties of high conduction speed, precise control of conduction speed, and invulnerability. Tonotopy is destroyed in mouse brain primary auditory cortex when the brain is demyelinated by administration of cuprizone (Cerina et al., 2017). The pathways that can comprise important cortical microcircuits fall into four classes. Besides the radial fibers, there are three types of horizontal pathways: the Exner stripe, the bands of Baillarger, and the late-myelinated U-fibers, which lie just outside the gray matter within the surface layer of the white matter. Two crucial questions can be raised in regard to each of these types of connection. The first is, are the fibers carrying inhibitory or excitatory signals? The second question relates to origins and destinations: are the fibers carrying signal traffic internal to each specific cortical area, or signals between separate cortical areas?

Generally speaking, at cortical area boundaries, the bands of Baillarger are discontinuous, changing their cortical depth from one area to the next within a fraction of the cortical thickness (as in Figure 3). Plainly, the axons comprising these bands play a much more important role within each cortical area and do not function as inter-areal connections. Less is known regarding the intra-areal and trans-areal connections of the other horizontal connections, although experimental techniques are available to investigate these questions.

## *In vivo* Visualization of Myeloarchitecture

It was recognized in the initial development of MRI that much of the contrast observed in brain tissue arose from the presence of myelin (for example Bydder and Steiner, 1982). Maps of the MRI parameters proton density and longitudinal relaxation time T1 each show a striking resemblance to myelin-stained sections of the brain. Initially, it was thought that T1 was dominated by the water content of the tissue, but that can be only part of the explanation because the fractional difference of T1 between gray and white matter (70%) is much larger than their ratio of water content (15%). NMR studies of model membranes made by Koenig (1991) and others in the 1990s revealed that T1 is strongly dependent on membrane composition. Essentially, the sink with which the spin magnetization of free water equilibrates after radiofrequency excitation, a process defining T1 relaxation, is comprised of the lipid content of the brain. The molecules cholesterol and cerebroside, in which myelin is rich, are particularly effective in T1 relaxation (Koenig, 1991; Kucharczyk et al., 1994). This finding has been confirmed unequivocally in studies (Leuze et al., 2017; Morawski et al., 2018) of the effect of lipid removal from brain tissue using CLARITY (Chung and Deisseroth, 2013) and related techniques. Clearing the lipid content, which includes all of the myelin sheaths except for a skeleton of hydrophilic proteins, entirely removes T1-based contrast between gray and white matter (Leuze et al., 2017). Independently, Stüber et al. (2014) showed that T1 measured in cadaver brain samples showed a linear dependence on myelin content, which was inferred from proton beam microscopy analysis of sections from the same tissue samples.

Although maps of T1 may not provide a fully quantitative measure of myelin content (see Callaghan et al., 2015; O’Muircheartaigh et al., 2019) and indeed depend slightly on the relative angle between axonal fiber direction and the static magnetic field (Knight et al., 2018), T1 is easy to measure accurately and quantitatively *in vivo* (Marques et al., 2010; Lutti et al., 2014), and thus offers itself as a reliable guide (Dubois et al., 2014) to the presence of myelin in brain tissue voxels, and as a quantitative means for comparing brains in longitudinal and cross-sectional studies. At magnetic fields of 7T and above, MRI has sufficient signal to noise ratio to resolve structures of less than 300 microns in size in an acceptable imaging time, provided that the effects of subject head motion are mitigated by prospective motion correction methods (e.g., Schulz et al., 2012). This has enabled the depiction of the entire primary visual cortex (Geyer et al., 2011), characterized by the well-defined Stria of Gennari (Gennari, 1782; Trampel et al., 2011), and several other cortical areas with prominent bands of Baillarger (Dinse et al., 2015; Fracasso et al., 2016). Figure 1B shows a coronal MRI section of occipital brain with good T1 contrast and 0.5 mm isotropic resolution, obtained in 2011 at 7T field strength. Several areas show cortical layering attributable to bands of Baillarger. Areas corresponding to those identified in the histological section are indicated in Figure 2B. At lower field strengths the spatial resolution is insufficient to discriminate cortical areas by means of their intensity profiles but still allows perhaps 15 myeloarchitectonically defined areas to be parcellated (Sereno et al., 2013).

Using the ratio of two MR image acquisitions with inverse T1 intensity weightings (one of these inappropriately named “T2-weighted”), Glasser and Van Essen (2011) were also able to discriminate several cortical areas. Techniques for mapping quantitative T1, and hence myelin density, continue to be rapidly developed by several groups. Of particular promise is a segmented simultaneous multi-slice inversion recovery echo-planar technique, MS-IR-EPI (Sanchez Panchuelo et al., 2018), which may provide 350 micron isotropic resolution images of volunteer subject brains with sharp definition of cortical myelin, given multiple imaging sessions and prospective motion correction to provide sufficient signal to noise ratio.

## Myeloarchitectonic Areas

The most definitive compilation of anatomically distinguishable cortical areas in human brain can be found, as already mentioned, in the work of Nieuwenhuys (2013), who analyzed the findings of Oscar and Cecile Vogt and their team, published in 20 articles between 1910 and 1954. These findings together provided a parcellation of the cortex (here termed the “Vogt parcellation”) into a total of 180 distinguishable areas, a figure reflected by the hybrid parcellation scheme of Glasser et al. (2016). The general picture is as follows: (a) primary brain areas, both sensory and motor, show a greater degree of myelination; (b) higher association areas in superior parietal cortex and prefrontal cortex have strikingly fewer myelinated axons, although no areas are devoid of either radial or horizontal myelinated axons; and (c) in those studies, such as Sanides (1964), which gave parcellations using both cytoarchitecture and myeloarchitecture, a precise congruence of boundaries was found.

*In vivo* myelin maps of the cortex using MRI are entirely consistent with these histological studies, although due to the lower SNR and poorer spatial resolution of MRI, only a small fraction of the Vogt parcellation has yet been unambiguously determined.

## Cortical Microcircuits

In the light of the observation that myelination is driven by a neuron’s experience, the uniformity of the myeloarchitecture within a cortical area carries a very interesting implication. A shared pattern of myelination may well imply shared experience. This suggests two linked hypotheses. The first is that each anatomically definable cortical area has a specific set of computational competencies—algorithms by which inputs to an area of cortex are transformed into outputs. The second hypothesis—which goes back to Flechsig’s early writings—is that stages in motor and cognitive development are reflected in episodes of more rapid myelination of specific cortical areas, as their axons myelinate and their neurons “come on stream,” thus becoming dramatically more effective participants in brain-wide neural networks. Cadaver brain studies of neonatal and infant myelination (Brody et al., 1987; Kinney et al., 1988) showed wide area-specific variations in the onset of myelination in short association fibers. Yeatman et al. (2014) have already shown that the MRI parameter T1, a quantitative measure of myelination, varies between white matter fascicles while remaining relatively uniform within each fascicle, and that each fascicle has a distinguishable longitudinal time course over the life span.

The hard wiring intrinsic to myelination surely results in privileged microcircuits specific to each cortical area. Techniques for modeling the performance of cortical microcircuits are well developed in the computational neuroscience literature (for example Potjans and Diesmann, 2014). Such microcircuits are protected from interference by unwanted additional synapse formation, they are made fast and durable, and the synchrony with which they carry action potentials is amenable to fine tuning using the adjustments available in the number of myelin layers and the internode spacing.

There are still few MRI studies of the relationship between cortical myelination and motor and cognitive performance. Ideally, such studies should be longitudinal, following a cohort of subjects over several years as they acquire new testable skills, such as reading. To resolve the bands of Baillarger, the highest possible spatial resolution would be needed, with 0.5 mm isotropic voxels or better, entailing the use of ultra-high field MRI of 7T or greater.

However, at lower resolution using 1.5 T MRI with 1 mm isotropic voxels, early work by Su et al. (2008) showed that the intensity of turbo-spin-echo images (erroneously described as “T2-weighted’) in language areas (including Broca’s Area) of the brains of children was correlated with their developmental stage. The results of Grydeland et al. (2013) at 3T using Glasser’s non-quantitative measure of myelin are also promising, showing a correlation of apparent net cortical myelination with performance in a speeded cognitive task. Kwon et al. (2018) also show the regional development of cortical myelination, although the inadequate technical standards of their study do not allow many important conclusions to be drawn; they use a non-quantitative myelin measure, unnecessary averaging of brain images across subjects, and excessive spatial smoothing, so that the details specific to actual cortical areas become entirely unobservable. Using MRI cortical maps of T1 in a group of infants between 3 and 21 weeks old, Lebenberg et al. (2019) identified five clusters of cortical areas with distinguishable development trajectories, confirming and extending the findings of Flechsig (1920). Very surprisingly, there is no mention of myeloarchitecture in this article, and no histological sections are shown. The level of superficial white matter myelination was found to correspond well to that of the adjacent cortex, suggesting that parallel processes take place within the cortex and in its white matter connections.

In a comprehensive study involving 484 participants, Grydeland et al. (2019) have explored the trajectory of myelination in more detail, showing waves of intracortical myelinogenesis and age-related demyelination, in which the age at which a plateau was reached was found to vary across the cortex. Age at peak growth had a bimodal distribution comprising a pre-pubertal wave of myelination in primary sensory and motor cortices, and a post-pubertal wave in association, insular and limbic cortices. The MR image resolution was insufficient in this study to allow greater specificity regarding the myeloarchitectonic areas involved in each of these waves.

A recent study (Andrews et al., 2017) examined differences in tissue contrast between gray and white matter in 98 adults diagnosed with autism spectrum disorder (ASD), and 98 typically developing controls. Although the authors were reticent regarding the interpretation of their data, the results indicated quite consistent hypermyelination of several cortical areas, mainly in the medial prefrontal region and in the ventral lateral temporal lobe. This suggests that a fundamental feature of autism is the premature maturation of a range of cortical microcircuits which cannot later be properly adapted for the processes required in social communication. This idea, which might provide further insights into the private world of the autistic brain, and indeed other refractory disorders that might be associated with structural slow learning processes, could be investigated using functional imaging that explored not only the responses to basic stimuli, but also feature spaces, especially the semantic feature space, and related this to the abnormalities in cortical myelination. Again, in the Andrews study, the spatial resolution was insufficient to enable the detailed analysis of cortical profiles needed to explore this further.

For evidence regarding the role of myelinated axons in cortical microcircuits, one must, therefore, turn to the relatively scanty cellular-level neuroscience studies using animal models. It is important to distinguish studies of horizontal and radial myelinated cortical axons. The recent work of Micheva et al. (2016) using tracer methods in mouse brain investigated the paths of myelinated horizontal cortical axons, making the important discovery that these interneurons mostly originate in basket cells, stain for parvalbumin, and are primarily inhibitory in their activity. This is inconsistent with earlier speculations that such axons are mainly collateral branches of excitatory axons of pyramidal cells in cortical layer IV (for example Braitenberg, 1962). This work has been replicated by Stedehouder et al. (2017). Micheva makes the crucial point that the myelination of horizontal cortical axons is unlikely to come about purely to increase their conduction velocity, because such axons are relatively short, and myelination would make very little difference to the transit time of an action potential. In corresponding work (2018) in human cortex, with cadaver brain samples from lightly myelinated cortical regions, Micheva et al. (2018) were able to confirm the presence of inhibitory myelinated parvalbumin axons, although with lower density than in mouse brain. It is worth commenting that if these workers had been able to study cortical areas with pronounced bands of Baillarger, they may have found a much higher proportion of inhibitory horizontal myelinated axons.

There has been a recent resurgence of interest in the neural connectivity of cortical Layer I. Here neuronal cell bodies are relatively scarce, and mostly GABA-ergic, but there is a dense neuropil consisting of dendritic arbors of pyramidal cells in deeper layers, and the horizontal myelinated axons of the Exner Stripe. Lam and Sherman (2018) review the scanty literature. In mouse brain, it appears that thalamocortical projections originating in nonspecific “thalamic matrix” nuclei and terminating in Layer I have the generic role of controlling and modulating cortical excitability (Cruikshank et al., 2012), a role which might explain the omnipresence of the Exner Stripe across the entire cortex. The results of Lam and coworkers suggested a wide range of modulatory control of Layer I neurons by metabotropic glutamate receptors and long range subcortical pathways.

Cauller et al. (1998) used a retrograde tracking method to investigate the paths of long horizontal fibers in Layer I of the rat somatosensory cortex, finding an isotropic distribution within the layer. In human cadaver brain primary visual cortex, however, using diffusion-weighted MRI, Leuze et al. (2014) provide evidence that the axons in Layer I have a preferred orientation within the layer, while the deeper myelinated layer known as the Stria of Gennari appears to show no preferential axonal orientation. Thus it remains unknown whether long myelinated fibers within Layer I link separate cortical areas, or form collaterals of thalamocortical axons, or simply serve to provide robust connections within a given cortical area.

In general, cellular neuroscience literature inexplicably fails to comment on the relatively prevalent myelination of horizontal axons lying in Layer I. Similarly, puzzlingly little research has ever been performed in regard to the functional role of the myelination of the axons comprising the horizontal bands of Baillarger, although this myelination is strikingly obvious anatomically, and energetically costly for the oligodendrocytes to produce. Few neuroanatomists have even been willing to speculate on the functional role of the Stria of Gennari in primary visual cortex. This feature, likely to consist largely of parvalbumin inhibitory axons arising from basket cells surrounding Layer IV pyramidal cells (Micheva et al., 2016), would seem to be a prime candidate both for the required “fast wipe” of the primary visual cortex needed to avoid the image streaking and blurring that would otherwise result from saccades, and for the well-established wide-scale center-surround inhibition that is driven by attentional focusing (Shmuel et al., 2006).

The appearance of the radial myelinated cortical axons is also highly characteristic for specific cortical areas (Vogt and Vogt, 1919), as Figure 1A clearly illustrates. Rowley et al. (2015) have already used the apparent depth of cortical myelin, visualized in relatively low-resolution MRI scans, as a crude index for cortical parcellation. Higher resolution structural MRI mapping of T1 is likely to provide better data for cortical profiling of myelin concentration, which will enable the histological details of specific myeloarchitectural areas to be associated unambiguously with the observed functional activity of each area in response to imposed tasks (Turner and Geyer, 2014; Turner, 2016).

The length of myelination of radial axons within gray matter is never more than about 2 mm, a small fraction of their typical myelinated length within white matter, so its presence cannot be easily explained by the need for rapid conduction velocity. Could the variability of this distance across cortical areas, together with the prevalence and degree of myelination of horizontal axons, be better explained by the varying needs between cortical areas for privileged microcircuits? To function effectively, some areas may require a high proportion of unmyelinated radial axons that in response to non-recurrent experience can easily participate in temporary synaptic connections to local dendritic arborizations, as needed for transitional computational operations. In mouse brain, many synapses have been observed to form and regress in a dynamic turnover (Loewenstein et al., 2015). On the other hand, other areas, such as primary motor cortex, may need very robustly defined output pathways that must not be modulated by extraneous connections.

## Can the Study of Myeloarchitectural Development be the Key for Understanding Cortical Microcircuits?

The foregoing remarks suggest the following interlinked research questions:

(1) Can high-resolution quantitative structural MRI (hqsMRI) at ultra-high field show the bands of Baillarger in many cortical areas, and thus provide an extensive structural parcellation?
(2) Does cortical myelination indeed proceed synchronously within each cortical area?
(3) Can hqsMRI track intracortical developmental changes in myelination?
(4) Do onsets or bursts of myelination in specific cortical areas coincide with transitions in cognitive development?
(5) Can components of the feature spaces revealed by machine learning analysis of functional imaging studies (such as voxel encoding Naselaris et al., 2011 or representational similarity Diedrichsen and Kriegeskorte, 2017) be associated with specific cortical areas?
(6) Can the hard wiring defined by the myelination pattern of each cortical area be used to model the operation of that area’s particular microcircuits?
(7) Can models based on such area-specific microcircuits predict the observed feature space components of that area?

The functional operation of cortical microcircuits may be experimentally observed using fMRI methods. Following the insights of Felleman and Van Essen (1991) and Callaway (2004), many studies (e.g., Harel et al., 2006) have used blood oxygenation level dependent (BOLD) and vascular space occupancy (VASO) fMRI with sufficiently high resolution to discriminate activity in different layers of the cerebral cortex (for a recent review, see Larkum et al., 2018). Pioneering work in 2010 by Polimeni (personal communication) showed that very high-resolution resting-state fMRI at 7T magnetic field could be used to investigate the correlation between activity in different cortical layers of separate cortical areas (in his work V1 and MT). This inspired later work (e.g., Huber et al., 2017; Trampel et al., 2017) which used layer-dependent fMRI together with experimental paradigms differentially emphasizing input and output activity in the same cortical area, in order to distinguish the roles of cortical input and output layers. Such experiments can infer causal directionality within brain networks at the systems level. Once the insights gained from detailed knowledge of local myeloarchitecture have been incorporated into such fMRI studies, the answers to the list of questions above may provide a dramatic advance in our understanding of human brain function.

## Author Contributions

RT wrote this entire article.

## Conflict of Interest Statement

The author declares that the research was conducted in the absence of any commercial or financial relationships that could be construed as a potential conflict of interest.
